# Implementation of MALDI-TOF Mass Spectrometry to Identify Fungi From the Indoor Environment as an Added Value to the Classical Morphology-Based Identification Tool

**DOI:** 10.3389/falgy.2022.826148

**Published:** 2022-03-15

**Authors:** Natacha Motteu, Berdieke Goemaere, Sandrine Bladt, Ann Packeu

**Affiliations:** ^1^Mycology and Aerobiology, Sciensano, Brussels, Belgium; ^2^Brussels Environment, Regional Intervention Cell for Indoor Pollution (RCIB/CRIPI), Brussels, Belgium

**Keywords:** indoor environment, microfungi, yeasts, identification, species, microscopy, MALDI-TOF MS

## Abstract

**Introduction:**

During the last decades, molds in the indoor environment have raised concern regarding their potential adverse health effects. The genera *Aspergillus, Cladosporium, Penicillium, Alternaria*, and yeasts, the most common fungi found indoors, include species with high allergenic and toxigenic potentials. Identification of these molds is generally performed by microscopy. This method has, however, some limitations as it requires mycologists with high expertise while identification is often limited to the genus level. Therefore, it is necessary to seek for fast and accurate tools, such as Matrix-assisted laser desorption/ionization time-of-flight mass spectrometry (MALDITOF MS), enabling an identification to the species level and guiding general practitioners in their search for the underlying cause of a health problem.

**Methods:**

In this study, 149 fungal air and dust isolates from 43 dwellings in Brussels were taken in collaboration with Brussels Environment RCIB/CRIPI and identified by both microscopy and MALDI-TOF MS in Sciensano's Indoor Mycology laboratory. Spectra obtained *via* MALDI-TOF MS were compared with data available in an in-house created reference database containing over 1,700 strains from the BCCM/IHEM fungal collection.

**Results:**

A total of 149 isolates including 18 yeasts and 131 filamentous fungi were analyzed. Microscopic analysis indicated 18 yeast species and allowed identification of 79 isolates (53%) to the genus level. Only 36 isolates (24%) could be identified to the species complex level. Fifteen molds (10%) could not be identified, and one was indicated as sterile mycelia. No isolate was identified to species level. MALDI-TOF MS analysis identified 137 (92%) of the 149 isolates with a logscore > 1.7. Of these 137 isolates, 129 (87%) were identified to the species level (logscore > 2.0). For only 8 isolates (5%), identification was limited to the genus/section level (1.7 < logscore <2.0), and 12 isolates (8%) could not be identified.

**Conclusion:**

A comparison of results obtained with both methods indicates an increased precision in identifications with MALDI-TOF MS analysis for 92% of the isolates, emphasizing its highly added value to the standard microscopic analysis in routine practice. In addition, MALDI-TOF MS also enables to assess the accuracy of microscopic identifications.

## Introduction

The quality of indoor air has become a subject of great interest as people are spending most of their time indoors (1). Biological pollutants such as fungi can, however, affect this air quality and can cause adverse health effects as fungal spores, mycotoxins, fungal allergens, and fungal volatile organic compounds can induce allergic, toxic or infectious effects (2, 3). The genera *Aspergillus, Cladosporium, Penicillium, Alternaria*, and yeasts are the most common fungi found indoors (4). A selected number of species within these genera have a high allergenic potential and/or can produce a significant amount of mycotoxins, capable to provoke adverse health effects in humans like allergic sinusitis, allergic rhinitis, allergic asthma, atopic dermatitis, hypersensitivity pneumonitis, or mycotoxicose (3, 5–7). In addition, some species within these genera are also capable of causing infections that can sometimes be invasive, such as candidemia or aspergillosis (7, 8). Besides the specificity of action and pathologies related to some specific molds, the patient's health background has to be taken into account as well while investigating adverse health effects that can be caused by mold problems indoors. In the case of immunocompromised or sensitized patients, an increased concentration of certain mold species indoors can represent an important health threat (6, 9).

Threats to health due to molds are a recurring and growing problem. This is partly due to the difficulty of determining an early diagnosis and, in the case of fungal infections, the resistance of many fungi to antifungal treatments (9, 10). Moreover, as health effects caused by molds can be dose-response related (11), it is very important to both determine the mold diversity as well as to quantify the load per species present indoors. In addition, in order to assess a potential indoor air mold contamination, the diversity of mold species and their quantities indoors must be compared to the outdoor situation (12). Therefore, the seek for fast and accurate tools enabling an identification to the species level of molds that could potentially threaten humans is important in order to guide general practitioners in their search for the underlying cause of a health problem and/or in applying the appropriate antifungal or allergic treatment.

Identification of molds found in the indoor environment is generally performed using microscopy. This standard method has, however, some limitations as it is time consuming and it needs mycologists with high expertise while identification is often limited to the genus level (2, 13). Molecular methods can offer relief (2, 14). However, DNA based techniques such as polymerase chain reactions are limited in the panel of species they can identify and multi-locus gene sequencing (the “gold standard” for the identification of microfungi) is very expensive, time consuming and prone to environmental contamination. An alternative method is matrix-assisted laser desorption/ionization time-of-flight mass spectrometry (MALDI-TOF MS), offering the advantage of being fast, easy to perform and cost-effective (15, 16). This analysis method has been used since the beginning of this century for the identification of bacteria, yeasts and molds (16). The principle of MALDI-TOF MS is based on the ionization of fungal proteins by a laser, followed by the creation of a spectrum, representing a species specific protein fingerprint. This spectrum is compared to a database of reference spectra and the similarity between the spectra is expressed in the form of a reliability index (“logscore”) (16–18).

In order to increase the accuracy of the fungal identifications, MALDI-TOF MS can be an added value to microscopy. In this study, the results of both microscopic and MALDI-TOF MS analyses will be compared to highlight the added value of MALDI-TOF MS for the identification of molds and to assess the accuracy of microscopic identifications.

## Materials and Methods

### Origin of the Strains and Preparation of the Samples

In collaboration with Brussels Environment RCIB/CRIPI (Regional Intervention Cell for Indoor Pollution), Sciensano's (Brussels, Belgium) Indoor Mycology unit performs measurements of fungal contaminations in Brussels dwellings in order to assess a link between a potential indoor air pollution and people's health problems. RCIB/CRIPI processes surveys in dwellings from people presenting a health issue, sampling indoor air, dust, settled dust on furniture and visible contaminated surfaces in various rooms, especially those where the patient spends a noticeable amount of time. In addition, the outdoor air is always sampled as a reference.

Air and dust samples were collected by the RCIB/CRIPI team between 28 May 2019 and 14 September 2021 during investigations in 43 different dwellings in Brussels. The air was sampled by a RCS+ impactor (volume of 80 L). Air samples were incubated for 5 days at 25°C and grown on HS culture medium (Rose Bengal agar + chloramphenicol, Biorad reference: R405.65.0100) to trace for the presence of mesophilic hygrophilic molds. Dust was sampled by a vacuum cleaner containing a filter (filter “3M filtrete” MC/US/diam 57 mm/PB) on a surface of 1 m^2^ during 2 min. Dust samples were suspended into a specific volume of Tween 80 (0.02%) solution depending on the amount of dust collected on the filter: the volume of Tween 80 (0.02%) in ml was calculated by multiplying the weight of dust 1000X (weights < 0.01 g) or 100x (weights > 0.01 g). After agitating the solution for 20 min, the primary solution was successively diluted 10 times. One ml of the solution of each sample was plated on MC (Malt extract agar + chloramphenicol, Biorad reference: 35M215.16) to search for mesophilic hygrophilic molds and another 1 ml of the solution of each sample was plated on M40Y + 10%NaCl (Malt extract – yeast extract – 40% sucrose agar + NaCl + chloramphenicol, Biorad reference: 35M222.65.0100) to search for xerophilic molds. Afterwards, the culture media were incubated for 7 days at 25°C.

### Morphological Analyses

After incubation, counting and macroscopic observation of the fungal colonies grown on the air strips and RODAC plates were performed by using a binocular magnifier (Leica MZ 6). The latter was followed by microscopic identification. Therefore, the colony to be investigated was taped with a small transparent adhesive tape or a small fragment was isolated and put between slide and slide coverslip and colored with lactophenol blue solution. Observation of the microscopic characteristics was performed with an optical microscope (Leitz Aristoplan).

### MALDI-TOF Analysis

For the colonies that could not be identified microscopically or in the case identification to the genus level or species complex level was not sufficient, a complementary MALDI-TOF MS analysis was performed.

In order to perform MALDI-TOF MS analysis, subcultures of 149 colonies were grown on SC culture medium (Sabouraud chloramphenicol agar) by incubation during 3-4 days at 25°C. Samples were prepared and analyzed following the method described by Becker et al. (19). After incubation, the strains were scratched delicately using disposable plastic forceps (Unomedical, Deeside, UK) and suspended each in a 1.5 ml Eppendorf tube (Eppendorf Safe-Lock tubes) containing 300 μl of sterile H_2_O. Each Eppendorf tube was then vortexed and 900 μl of absolute ethanol was added. After a centrifugation of 10 min at 1,300 rpm, the supernatant was discarded and the Eppendorf tube was air dried for 30 min. Then, 50 μl of formic acid 70% was added to the pellet and each tube was mixed with a pipette tip to resuspend the pellet material. After 5 min, 50 μl of acetonitrile 100% was added and followed by a vortexing step. The Bruker Bacterial Test Standard (BTS, Bruker Daltonics) was used for calibration (positive control). Each sample was centrifuged for 2 min at 13,000 rpm and 1 μl of the supernatant of each extract was then spotted onto a polished steel target plate (Bruker Daltonics) in quadruplicate, and dried at room temperature. Thereafter, 1 μl of matrix HCCA (α-Cyano-4-hydroxycinnamic acid) was added on all the spots containing the samples, on the positive control and a spot with only matrix was used as negative control. The Maldi-TOF MS analyses were performed with the Microflex LT MALDI-TOF MS system and MALDI Biotyper Compass Explore software of Bruker Daltonics.

The database of references spectra used in this study is an in-house created reference database containing over 2,790 strains of the BCCM/IHEM fungal collection (https://msi.happy-dev.fr/login/) representing 1,370 species and 360 genera, amongst which it includes 20 *Alternaria* species, 25 *Cladosporium* species, 142 *Penicillium* species, 46 *Talaromyces* species, 164 *Aspergillus* species and 280 yeasts species. All reference strains' identities were confirmed by performing DNA sequencing. A control of the specificity of their main spectra was performed by challenging a subculture of each reference strain against the database. The identities of the strains used to make these challenges were preliminarily controlled, and they were not the same strains as those used to build the database.

The spectra obtained by MALDI-TOF MS analyses performed on our samples were compared with the in-house created reference database of the BCCM/IHEM fungal collection. Logscore values were provided to express the similarity between the obtained spectra and the reference spectra from the database. According to the manufacturer, a logscore < 1.7 corresponds to an unreliable identification, a logscore between 1.7 and 1.99 indicates acceptable genus identification while a logscore equal to or above 2.0 indicates acceptable species level identification (15).

## Results

A total of 149 isolates, including 18 yeasts and 131 filamentous fungi, were analyzed.

Microscopic analysis indicated 18 isolates as yeasts and allowed identification to the genus level for 79 microfungi. Only 36 isolates could be identified to the species complex level. Fifteen could not be identified nor to the genus nor to the species (complex) level and 1 was indicated as sterile mycelia (Table 1).

**Table 1:** Summary comparing identifications performances of microscopy and MALDI-TOF MS analyses.

**149 isolates analyzed**	**Microscopy**	**MALDI-TOF MS**
Genus level	79 isolates (53%)	8 isolates (5%)
Species complex level	36 isolates (24%)	/
Species level	/	129 isolates (87%)
Other	18 yeasts, 1 sterile mycelia, 15 isolates not identified (10%)	12 isolates not identified (8%)

In comparison, analysis using MALDI-TOF MS identified 137 of the 149 isolates with a logscore > 1.7. Of these 137 isolates, 129 were identified to the species level with a logscore > 2.0. For 8 isolates, identification was limited to the genus/section level with a logscore between 1.7 and 2.0 and 12 isolates could not be identified (Table 1).

The results of the identifications by both methods are listed in Table 2.

**Table 2:** Identifications of the isolates by MALDI-TOF MS vs. microscopy, including origin of isolates.

**Genus/species complex**	**MALDI-TOF MS identification (number of isolates)**	**Microscopic identification (number of isolates)**	**Isolate origin**
***Penicillium***			
*Penicillium* species	*Penicillium chrysogenum* (13), *Penicillium crustosum* (11), *Penicillium brevicompactum* (7), *Penicillium rubens* (5), *Penicillium frequentans* (3), *Penicillium citrinum* (3), *Penicillium camemberti* (2), *Penicillium fellutanum* (2), *Penicillium bialowiezense* (2)*, Penicillium polonicum* (2), *Penicillium atramentosum* (2), *Penicillium griseofulvum* (1), *Penicillium olsonii* (1), *Penicillium echinulatum* (1), *Penicillium biforme* (1), *Penicillium camemberti* gr (1), *Penicillium* species (section *aspergilloides*) (1), *Penicillium* species (*brevicompactum* gr.?) (1)	*Penicillium* species (59)	Indoor air
	*Penicillium polonicum* (1)	*Penicillium* species (1)	Dust
***Cladosporium***			
*Cladosporium herbarum* gr.	*Cladosporium allicinum* (8)	*Cladosporium herbarum* gr. (7), *Cladosporium* species (1)	Indoor air
	*Cladosporium aggregatocicatricatum* (2)	*Cladosporium herbarum* gr. (2)	Indoor air
	*Cladosporium ramotenellum* (2)	*Cladosporium herbarum* gr. (1), *Cladosporium* species (1)	Indoor air
*Cladosporium cladosporioides* gr.	*Cladosporium westerdijkiae* (1)	*Cladosporium herbarum* gr. (1)	Indoor air
	*Cladosporium inversicolor* (1)	*Cladosporium* species (1)	Indoor air
	*Cladosporium delicatulum* (4)	*Cladosporium cladosporioides* gr. (2), *Cladosporium sphaerospermum* gr. (2)	Indoor air
	*Cladosporium cladosporioides* (2)	*Cladosporium cladosporioides* gr. (1), *Cladosporium* species (1)	Indoor air
	*Cladosporium pseudocladosporioides* (1)	*Cladosporium cladosporioides* gr. (1)	Indoor air
	*Cladosporium europaeum* (1)	*Cladosporium sphaerospermum* gr. (1)	Indoor air
*Cladosporium sphaerospermum* gr.	*Cladosporium halotolerans* (1)	*Cladosporium* species (1)	Indoor air
	*Cladosporium sphaerospermum* (2)	*Cladosporium sphaerospermum* gr. (1), *Cladosporium* species (1)	Indoor air
***Aspergillus***			
*Aspergillus fumigatus* gr.	*Aspergillus fumigatus* (3)	*Aspergillus fumigatus* gr. (3)	Indoor air
*Aspergillus versicolor* gr.	*Aspergillus creber* (3), *Aspergillus sydowii* (2)	*Aspergillus versicolor* gr. (5)	Indoor air
*Aspergillus flavus* gr.	*Aspergillus flavus* (4)	*Aspergillus flavus* gr. (3), *Aspergillus ochraceus* gr. (1)	Indoor air
*Aspergillus niger* gr.	*Aspergillus tubingensis* (3)	*Aspergillus niger* gr. (1), *Aspergillus* species (2)	Indoor air
*Aspergillus glaucus* gr.	*Aspergillus pseudoglaucus* (2)	*Aspergillus glaucus* gr. (1), *Aspergillus* species (1)	Indoor air
*Aspergillus ochraceus* gr.	*Aspergillus persii* (1)	*Aspergillus flavus* gr. (1)	Indoor air
*Aspergillus nidulans* gr.	*Aspergillus nidulans* (1)	*Aspergillus* species (1)	Indoor air
*Aspergillus restrictus* gr.	*Aspergillus restrictus* (2)	*Aspergillus restrictus* gr. (2)	Dust
*Aspergillus ustus* gr.	*Aspergillus calidoustus* (1)	Not identified (1)	Indoor air
	*Aspergillus* species (section *nidulantes*) (1)	*Aspergillus* species (1)	Indoor air
**Yeasts**			
	*Rhodotorula mucilaginosa* (4), *Debaryomyces hansenii* (3), *Saccharomyces cerevisiae* (3), *Naganishia diffluens* (3), *Candida parapsilosis* (1), *Starmerella etchellsii* (1)	Yeast species (15)	Dust
	*Rhodotorula mucilaginosa* (1)	Yeast species (1)	Indoor air
**Other species**			
	*Pseudopithomyces* species (1)	*Alternaria* species (1)	Indoor air
	*Hormographiella verticillata* (*Caprinellus domesticus*) (1)	Not identified (1)	Indoor air
	*Exophiala sideris* (1)	Not identified (1)	Dust
	*Plectosphaerella cucumerina* (1)	Not identified (1)	Indoor air
	*Talaromyces Wortmannii* (1)	*Paecilomyces* species (1)	Indoor air
	*Botrytis cinerea* (1)	*Botrytis* species (1)	Indoor air
	*Parengyodontium album* (1)	Not identified (1)	Indoor air
	*Parengyodontium* species (1)	Not identified (1)	Indoor air
	*Beauveria bassiana* (1)	Not identified (1)	Indoor air
	*Microascus croci* (1)	Not identified (1)	Indoor air
	*Acrodontium crateriforme* (1), not identified (2)	*Acremonium* species (3)	Indoor air
	*Acrodontium species* (1)	Not identified (1)	Indoor air
	*Calcarisporium* species (1)	*Acremonium* species (1)	Indoor air
	Not identified (8)	Sterile mycelia (1), *Penicillium* species (1), yeast species (2), not identified (4)	Indoor air
	Not identified (2)	Not identified (2)	Dust

Compared to microscopic analysis, MALDI-TOF MS resulted in a more precise identification for 137 isolates (92%). Of the 97 isolates identified microscopically to the genus level or as belonging to yeasts and of the 36 isolates identified microscopically to the species complex level, respectively 90 and 30 isolates were confirmed as correctly identified when compared to the results obtained by MALDI-TOF MS analysis. The 30 isolates identified by microscopy to the species complex level and indicated as correct by MALDI-TOF MS included 15 *Cladosporium* isolates (10 *Cladosporium herbarum* gr., 4 *Cladosporium cladosporioides* gr. and 1 *Cladosporium sphaerospermum* gr.) and 15 *Aspergillus* isolates (5 *Aspergillus versicolor* gr., 3 *Aspergillus fumigatus* gr., 3 *Aspergillus flavus* gr., 1 *Aspergillus glaucus* gr., 1 *Aspergillus niger* gr. and 2 *Aspergillus restrictus* gr.). Microscopic identification of the 60 *Penicillium* isolates was limited to the genus level whereas yeasts were only referred to as “yeast species.” In contrast, MALDI-TOF MS indicated a diversity of 15 different *Penicillium* species and 6 different yeasts species. Two isolates identified microscopically as belonging to yeasts and one isolate identified microscopically as *Penicillium* species could not be identified by MALDI-TOF MS (Table 2).

Of the 15 isolates that could not be identified by microscopy, MALDI-TOF MS identified 7 isolates to the species level (*Hormographiella verticillata, Exophiala sideris, Plectosphaerella cucumerina, Parengyodontium album, Beauveria bassiana, Microascus croci*, and *Aspergillus calidoustus*) and 2 to the genus level (*Parengyodontium* species and *Acrodontium* species) (Table 2). Six microscopic misidentifications to the species complex level were observed. Three isolates identified as *Cladosporium sphaerospermum* gr. by microscopy were identified by MALDI-TOF MS as two *Cladosporium delicatulum* isolates and one *Cladosporium europaeum* isolate, both members of the *Cladosporium cladosporioides* species complex. Another isolate, microscopically identified as *Cladosporium herbarum* gr., was identified as *Cladosporium westerdijkiae* (*Cladosporium cladosporioides* gr.) with MALDI-TOF MS. Within the genus *Aspergillus*, 2 microscopic misidentifications occurred and were correctly identified using MALDI-TOF MS: an *Aspergillus ochraceus* gr. isolate turned out to be *Aspergillus flavus* and an isolate of *Aspergillus flavus* gr. was identified as *Aspergillus persii* (*Aspergillus ochraceus* gr.) (Table 2).

In addition, 1 microscopically identified *Alternaria* isolate was re-identified as *Pseudopithomyces* species with MALDI-TOF MS analysis, one isolate identified as *Paecilomyces* species was reidentified as *Talaromyces wortmannii* according to MALDI-TOF MS and 2 isolates microscopically identified as *Acremonium* species were identified with MALDI-TOF MS as *Acrodontium crateriforme* and *Calcarisporium* species (Table 2).

## Discussion

A comparison between identifications performed by microscopic analysis and MALDI-TOF MS analysis highlights their difference in accuracy of identification. Microscopic analysis did not allow an identification to the genus level for none of the yeast isolates and more than half of all microscopic identifications in this study (53%) were limited to an identification to the genus level. Less than one quarter of the isolates (24%) could be identified to the species complex level by observation of morphological characteristics. These findings highlight the difficulty of species differentiation within genera and within species complexes based on morphological characteristics. Indeed, several species might have similar morphological characteristics and species within most genera of molds are often hard to differentiate, especially when it comes to specific structures such as spores (13, 20). Our results show that *Penicillium* isolates can hardly be identified to the species level and yeasts cannot be further identified by the standard microscopic method, as also highlighted by Barton (21), Reboux et al. (22), and Robert et al. (20).

In contrast, MALDI-TOF MS analysis allowed the identification to the species level for 87% of the isolates analyzed, which is consistent with the results obtained in several studies using the Brucker BioTyper system (16). MALDI-TOF MS enabled insight into the diversity of *Penicillium* found in the sampled dwellings. Of all *Penicillium* isolates identified by MALDI-TOF MS analysis, 60% belonged to 4 species: *Penicillium chrysogenum* (21.6%), *Penicillium crustosum* (18.3%), *Penicillium brevicompactum* (11.6%) and *Penicillium rubens* (8.3%). This is consistent with the study of Reboux et al. (22) aiming to identify indoor air and dust *Penicillium* species by MALDI-TOF MS and molecular methods, where *P. chrysogenum* and *P. rubens* together accounted for 27.3% of all *Penicillium* species identified indoors. Indeed, *P. chrysogenum* is often put forward as the most common *Penicillium* species found in dwellings (22). *Penicillium chrysogenum* is moreover considered as an important cause of allergic reactions and *P. crustosum* and *P. brevicompactum* are also able to produce allergens (3, 6). In addition, *P. chrysogenum* and *P. brevicompactum* are both also capable of producing numerous mycotoxins (5, 22). As for the results of *Penicillium* species, MALDI-TOF MS analysis also demonstrated a wide diversity of yeasts species found in the indoor environment of the 43 Brussels dwellings analyzed in this study. *Rhodotorula mucilaginosa* was the most dominant yeast species, followed by *Naganishia diffluens, Debaryomyces hansenii* and *Saccharomyces cerevisiae*, the latter three being equally present. Of them, *S. cerevisiae* can be implicated in allergic reactions such as atopic dermatitis and *Naganishia diffluens* can be an exacerbating factor for this reaction (3, 23). *Rhodotorula mucilaginosa* is also able to produce allergens (3) while *Debaryomyces hansenii* seems to be rarely associated with health problems in humans (8).

In addition, several microscopic misidentifications occurred at the level of the species complexes, i.e., between *Aspergillus flavus* and *Aspergillus ochraceus*, and between the species complexes of *Cladosporium herbarum, C. cladosporioides* and *C. sphaerospermum*. Moreover, as for *Penicillium*, some *Aspergillus* and *Cladosporium* isolates could not be identified further than the genus level by microscopy. *Cladosporium herbarum* gr. and *C. cladosporioides* gr. accounted for 88% of all *Cladosporium* isolates analyzed in this study while *C. sphaerospermum* gr. occurred in 12% of the samples. These results are in accordance with the findings of Segers et al. (24), who identified *C. sphaerospermum* gr. as the less frequent *Cladosporium* species complex found in indoor air. Four isolates of the species complex *C. cladosporioides* were microscopically misidentified, one as the species complex *C. herbarum* and three as *C. sphaerospermum* gr. Although all three species complexes are known to produce allergenic proteins (5, 6), their relative presence is often not equal in indoor and outdoor environments, especially not in poorly ventilated houses (24). Therefore, an accurate identification of *Cladosporium* to the species level is very important in order to trace for a potential indoor contamination. Considering *Aspergillus*, as both allergic and toxic reactions are mainly caused by *Aspergillus fumigatus, Aspergillus flavus, Aspergillus niger* and *Aspergillus versicolor* (3, 5, 6, 25), an accurate identification to the species level is of high importance for this genus as well.

Some environmental isolates (8%) could not be identified by MALDI-TOF MS analysis, which could be explained by the predominance of spectra from clinical human isolates and the lack of environmental isolates in the current MALDI-TOF MS databases (including our in-house database). In order to strengthen this identification tool for indoor and outdoor environmental isolates, our in-house created database is currently being expanded with spectra of environmental isolates by performing DNA extraction and sequencing on isolates that could not be identified with MALDI-TOF MS analysis.

Nowadays the lack of extended reference spectra databases that are commercially available is one of the major limitations of MALDI-TOF MS identifications of yeasts and especially of microfungi, as stated by Hendrickx (16), Sanguinetti and Posteraro (26), and Robert et al. (20). This can explain why numerous fungal identifications by MALDI-TOF MS are based on the use of in-house created databases (16). It has been shown by studies summarized in Sanguinetti and Posteraro (26) that MALDI-TOF MS analyses based on in-house created databases reach a significantly higher identification rate. This highlights that the capacity of identification is highly dependent on the quality of the database used in the laboratories, i.e., notably the number of species and quantity of spectra available per species in the database, the quality of the spectra, … (20). Therefore, it seems that databases should be constantly updated and extended with new encountered fungal and yeasts species in order to improve the species coverage of MALDI-TOF MS reference databases, which is currently still a limitation, as noted by Bader (27), Becker et al. (19), and Sanguinetti and Posteraro (26).

Moreover, the availability of public databases is also a concern. Making the created in-house databases publically available could help to enhance the identification rates. However, this would mean that protocols used to perform the analyses would have to be standardized and available for the scientific community. Indeed culture conditions, sample preparation, extraction method and products and the spectrometer used can be an important source of variability and influence the success rate of correct identification (16, 18, 28). In order to overcome the latter, besides the availability of one common commercially available open access database, a standardization of the protocol could be a big step forward in the optimization of MALDI-TOF MS analyses.

Another limitation is the incubation time of samples on solid culture media and subcultures of isolates preliminary to MALDI-TOF MS analysis. To overcome the limitations inherent to MALDI-TOF MS analysis, innovative air sampling techniques collecting biological particles into liquid medium could be considered. The fungal DNA could be extracted from the liquid medium avoiding the time consuming culture step. PCR followed by sequencing could then be used to identify the species diversity. However, this technique does not allow for a quantification of the different species present in the samples, although this being very important as explained earlier. In order to overcome the latter, qPCR can be considered but good mold-specific target genes can be difficult to find and determine. In addition, DNA-based methods are expensive and can be hard to implement in laboratory routine analyses (20).

The results of our study demonstrate the great capacity of MALDI-TOF MS in identifying fungal air and dust isolates to the species level. Indeed, MALDI-TOF MS appears to be an added-value to the standard microscopy technique in identifying molds as it can enable the differentiation between closely related and cryptic species in comparison with conventional methods (16). In addition, MALDI-TOF MS also allows to gain new epidemiological insights linked to environmental indoor mold pollution. In contrast to morphological observations, MALDI-TOF MS is an automated tool, yielding results with a high objectivity level. In addition, the accuracy of MALDI-TOF MS can be increased here by applying our in-house created reference database, containing only highly controlled fungal strains from the BCCM/IHEM collection, both ISO 9001 certified and ISO 17025 accredited (15, 19). These findings make MALDI-TOF MS a highly added value to microscopy for the identification of fungal isolates in routine analyses in our laboratory.

## Conclusion

A comparison between identifications of fungal isolates performed by microscopic analysis and MALDI-TOF MS analysis highlights the higher accuracy of the latter. The identifications to the species level with MALDI-TOF MS analysis enable the comparison of species diversity indoors vs. outdoors in order to assess a potential indoor mold contamination. Moreover, it can help to guide general practitioners in their search for the underlying cause of a health problem and/or in applying the appropriate antifungal or allergic treatment.

The automated and objective MALDI-TOF MS tool can be a highly added value to the time consuming standard microscopic analysis in routine practice aiming to identify molds from dwellings. However, MALDI-TOF MS databases, being mostly built on spectra from clinical human isolates, should be extended by including environmental isolates in order to strengthen the identification tool. Innovative sampling techniques combined with DNA analyses can offer interesting perspectives in the identification of molds present in indoor environment samples. However, these techniques remain expensive and difficult to implement in laboratory routine practices.

## Data Availability Statement

The original contributions presented in the study are included in the article/supplementary material, further inquiries can be directed to the corresponding author/s.

## Author Contributions

NM: design of the work, data analysis and interpretation, drafting the article, and critical revision of the article. BG: data interpretation, drafting the article, and critical revision of the article. AP: critical revision of the article. SB: data collection and critical revision of the article. All authors contributed to the article and approved the submitted version.

## Funding

This work was supported by Sciensano (Brussels, Belgium) and Brussels Environment (Brussels, Belgium).

## Conflict of Interest

The authors declare that the research was conducted in the absence of any commercial or financial relationships that could be construed as a potential conflict of interest.

## Publisher's Note

All claims expressed in this article are solely those of the authors and do not necessarily represent those of their affiliated organizations, or those of the publisher, the editors and the reviewers. Any product that may be evaluated in this article, or claim that may be made by its manufacturer, is not guaranteed or endorsed by the publisher.
